# *EHMT1* mosaicism in apparently unaffected parents is associated with autism spectrum disorder and neurocognitive dysfunction

**DOI:** 10.1186/s13229-018-0193-9

**Published:** 2018-01-25

**Authors:** Anneke de Boer, Karlijn Vermeulen, Jos I. M. Egger, Joost G. E. Janzing, Nicole de Leeuw, Hermine E. Veenstra-Knol, Nicolette S. den Hollander, Hans van Bokhoven, Wouter Staal, Tjitske Kleefstra

**Affiliations:** 1 Karakter Child and Adolescent Psychiatry University Centre, Nijmegen, the Netherlands; 2 Donders Institute for Brain, Cognition and Behavior, Centre for Neuroscience, Radboud University, Nijmegen, the Netherlands; 3 Department of Psychiatry, Radboud University Medical Center, Nijmegen, the Netherlands; 4 Centre of Excellence for Neuropsychiatry, Vincent van Gogh Institute for Psychiatry, Venray, the Netherlands; 5 Behavioural Science Institute, Radboud University, Nijmegen, the Netherlands; 6 Donders Institute for Brain, Cognition and Behaviour, Centre for Cognition, Radboud University, Nijmegen, the Netherlands; 7 Department of Human Genetics, Radboud University Medical Center, P.O. Box 9101, 6500 HB Nijmegen, the Netherlands; 8 Department of Genetics, University Medical Centre Groningen, University of Groningen, Groningen, the Netherlands; 9 Department of Clinical Genetics, Leiden University Medical Center, Leiden, the Netherlands; 10 Faculty of Social Sciences, Leiden University, Leiden, the Netherlands; 11 Leiden Institute for Brain and Cognition, Leiden University, Leiden, the Netherlands

**Keywords:** Kleefstra syndrome, *EHTM1*, Mosaicism, Cognition, Autism spectrum disorder, Major depressive disorder

## Abstract

**Background:**

Genetic mosaicism is only detected occasionally when there are no obvious health or developmental issues. Most cases concern healthy parents in whom mosaicism is identified upon targeted testing of a genetic defect that was initially detected in their children. A germline genetic defect affecting the euchromatin histone methyltransferase 1 (*EHMT1*) gene causes Kleefstra syndrome, which is associated with the typical triad of distinct facial appearance, (childhood) hypotonia, and intellectual disability. A high degree of psychopathology is associated with this syndrome. A few parents with a mosaic *EHMT1* mutation have been detected upon testing after a child was diagnosed with a germline *EHMT1* defect. At first glance, carriers of a mosaic *EHMT1* mutation appeared to function normally. However, recent studies have shown that de novo, postzygotic mutations in important developmental genes significantly contribute to autism spectrum disorder (ASD). Therefore, we hypothesized that *EHMT1* mosaicism could cause neuropsychiatric defects. To investigate this, we performed a detailed investigation of cognitive neuropsychiatric parameters in parents identified with *EHMT1* mosaicism.

**Methods:**

Three adults (two males, one female) with a genetically confirmed diagnosis of *EHMT1* mosaicism were examined by means of a battery of tests and observational instruments covering both neurocognitive and psychiatric features. The battery included the following instruments: the Autism Diagnostic Observation Schedule (ADOS), the mini Psychiatric Assessment Schedules for Adults with Developmental Disabilities (mini PAS-ADD), the Vineland Adaptive Behavior Scales (VABS), and the Cambridge Neuropsychological Test Automated Battery (CANTAB). These measures were compared with our previously reported data from Kleefstra syndrome patients with confirmed (germline) *EHMT1* defects.

**Results:**

All three subjects achieved maximum total scores on the VABS, indicative of adequate (adaptive) functioning. In all, scores above cutoff were found on the ADOS for ASD and on the mini PAS-ADD for major depressive disorder (lifetime). Finally, results on the CANTAB showed impaired cognitive flexibility in all subjects.

**Conclusion:**

Individuals with *EHMT1* mosaicism seem to have increased vulnerability for developing severe psychopathology, especially ASD and mood disorders. Although at first glance they appear to be well-adapted in their daily functioning, they may experience significant psychiatric symptoms and show reduced cognitive flexibility in comparison to the general population.

## Background

Kleefstra syndrome (KS; OMIM #610253) is caused by haploinsufficiency of the *euchromatin histone methyltransferase* 1 (*EHMT1*) gene located at chromosome 9q34.3. Almost all cases of KS are due to de novo microdeletions or intragenic loss-of-function mutations in this gene [1, 2]. So far, no inherited cases have been reported, except for a few cases on the basis of parental mosaicism. Mosaicism for *EHMT1* defects appears to be rare. At present, only four cases have been reported: three with a 9q34.3 microdeletion [3, 4] and one with a splice-site mutation in *EHMT1* [5]. These carriers of a genetic *EHMT1* mosaicism were reported to function normally in daily life. However, the focus of these reports was on the genetic and somatic features of the subjects. No data was presented on the possible psychopathology, which is the most pronounced comorbidity in patients with KS caused by a germline *EHMT1* defect [6–9].

The full KS phenotype is characterized by the core triad of intellectual disability (ID), (childhood) hypotonia, and distinct facial features. Additional clinical features include specific behavioral characteristics, heart and urogenital defects, epilepsy, and overweight [1, 2, 10]. Recently, we studied the specific profile of behavioral characteristics in KS, which led us to conclude that KS patients are extremely vulnerable to developing severe psychiatric disorders. In our cohort, the prevalence of autism spectrum disorder (ASD) is nearly 100% [6]. In addition, we found high prevalence of mood disorders and psychotic disorders. Moreover, patients above the age of 18 years in our cohort showed severe regression with a loss of at least 30–50% of their initial functioning [6, 8, 11]. Interestingly, recent studies have shown that de novo postzygotic mutations (PZM) in important developmental genes contribute to ASD [12, 13]. Based on this evidence, we set out to investigate the presence of psychopathology in the parents with an *EHMT1* mosaicism.

In this study, we examined three subjects with *EHMT1* mosaicism for the presence of psychopathology. All three subjects are seemingly unaffected parents of children diagnosed with KS caused by a germline *EHMT1* defect. We hypothesized that individuals with *EHMT1* mosaicism are vulnerable to psychopathology which may have significant clinical consequences.

## Methods

### Participants

Subjects with *EHMT1* mosaicism were invited by the Department of Human Genetics, Radboud University Medical Center, Nijmegen, the Netherlands, to participate in this study. Informed consent was obtained. The regional medical ethical committee (medical research ethics committee CMO/METC Arnhem-Nijmegen, the Netherlands) approved the study (NL43187.091.13), which was performed in full accordance with the Declaration of Helsinki.

Subject characteristics are summarized in Table 1.

**Table 1:** Genetic, psychopathological, and neurocognitive subject characteristics of the subjects

	Subject 1	Subject 2	Subject 3
Sex/age (years)	Male/44	Female/43	Male/39
Genetic defect [*EHMT1* mosaicism]	∼ 60 kb deletion exons 5–17 *EHMT1* 40% mosaicism in blood lymphocytes and buccal swab	∼ 200 kb deletion exons 1–5 *EHMT1* 80% mosaicism buccal swab	∼ 200 kb deletion exon 1 *EHMT1* 60% mosaicism blood lymphocytes
VABS (maximum score)			
Communication (134)	134	121	132
Daily living skills (184)	184	176	177
Socialization (132)	132	118	132
Raw score (450)	450	415	441
Mean developmental age in years	> 12	> 12	> 12
ADOS module 4 (cutoff score)			
Communication (2)	4	2	2
Social interaction (4)	7	10	9
Total score (7)	11	12	11
Classification	Autism spectrum disorder	Autism spectrum disorder	Autism spectrum disorder
Mini PAS-ADD			
Major depressive disorder	Past	Present and past	Present and past
Anxiety disorder	–	Present and past	–
(Hypo)mania	–	–	–
Obsessive compulsive disorder	–	Present and past	–
Psychosis	–	Present and past	–
Unspecified disorder	–	–	–
Autism spectrum disorder	–	–	–
CANTAB *			
MOT mean latency	85–90%	40–45%	80–85%
MOT error	70–75%	95–100%	40–45%
PRM	20–25%	0–5%	70–75%
IED error	30–35%	10–15%	10–15%
IED completed stages	15–20%	5–10%	10–15%
PAL	55–60%	0–5%	75–80%

*VABS* Vineland Adaptive Behavior Scales, *mini PAS-ADD* Psychiatric Assessment Schedules for Adults with Developmental Disabilities, *CANTAB* Cambridge Neuropsychological Test Automated Battery, *MOT* Motor Screening Test, *PRM* Pattern Recognition Memory, *IED* Intra-Extra Dimensional, *PAL* Paired Associate Learning

*The percentages represent the subjects’ performance compared to the normative group. For example, a subject score of 10% means that 90% of the normative group has better scores on this item

### Mosaic pattern detection

In all three subjects, the presence of mosaicism was detected upon carrier testing after the initial finding of a deletion in 9q34 by genome-wide arrays in their respective children. Subsequent confirmation by fluorescent in situ hybridization (FISH) and/or multiplex ligation-dependent probe amplification (MLPA) in blood or in additional tissue was performed in all three subjects.

### Instruments

The instruments that were used in this case study are briefly described below. More extensive details can be found in the study of Vermeulen et al. [6] where the instruments have been applied previously in a large cohort of patients with the full KS phenotype.

The Autism Diagnostic Observation Schedule (ADOS) is a semi-structured observational method to assess autism features [14, 15]. It is performed by a certified psychologist or psychiatrist (in this cohort, the first author, KV) and consists of four modules, based on the (developmental) age and language capacity of the participant. Module 4 is designed for normal functioning adults and was used in the current study. A total cutoff score of 7 and above is suspect for an ASD.

The Dutch version of the mini Psychiatric Assessment Schedules for Adults with Developmental Disabilities (mini PAS-ADD) is a psychometrically adequate interview for the detection of psychiatric symptoms and disease in persons with developmental disabilities [16, 17]. It consists of 86 items on a 4-point scale: 0 (symptom not present) to 3 (symptom is severe). The interview is divided into seven subscales: major depressive disorder (MDD), anxiety, obsessive-compulsive disorder, hypomania/mania, psychosis, unspecified disorder, and autism. All criteria are based on the International Classification of Diseases (ICD-10). In this study, subjects were interviewed about themselves.

The Dutch adaptation of the Vineland Adaptive Behavior Scales (VABS) [18] is a widely used clinical interview, which determines the level of adaptive functioning. The age-equivalent scores aid in classifying intellectual and developmental disabilities. The VABS consists of three domains: communication skills, daily living skills, and social skills. This instrument has good reliability and validity [19]. In this study, the subjects were interviewed about themselves.

The Cambridge Neuropsychological Test Automated Battery (CANTAB) is a tablet-based neuropsychological test set comprising all major cognitive domains. Four subtests of the CANTAB were performed in the following sequence:

1. The Motor Screening Test (MOT) is a training procedure designed to introduce the subject to the tablet touchscreen. In our cohort, it is also an estimate to reactivity, which could not be tested with an official reaction time test because of the complexity of the latter (pushing a button instead of the touch screen during the task).
2. The Pattern Recognition Memory (PRM) is a test of visual pattern recognition memory in a two-alternative forced choice discrimination paradigm. These patterns are designed in such a way so that they cannot easily be given verbal labels. This study presents the number of correct responses.
3. The Intra-Extra Dimensional Test Shift (IED) is a test of rule acquisition and reversal. Visual discrimination, cognitive flexibility, and sustained attention are tested. This study presents (1) total errors, a measure of the subject’s efficiency in attempting the test, and (2) the number of completed stages.
4. Paired Associate Learning (PAL) assesses visual memory and new learning. It consists of eight stages. Each stage is composed of several trials, consisting of the first presentation of the shape(s) and followed by representation when the subject makes an error. The clinical mode terminates after ten repeat presentations. This study presents the total number of errors.

### Procedure

Subjects were visited at home. After obtaining informed consent, the study procedure consisted of two clinical interviews with the subjects (VABS and mini PAS-ADD), followed by a semi-structured observation (ADOS) and the tablet-based neurocognitive tests (CANTAB). These procedures were performed by a certified psychiatrist (KV).

## Clinical reports

Subject 1 is a 44-year-old married male. He is a father of two children; the youngest son was diagnosed with KS. After finishing a higher professional education, he started working as a data engineer. He does not report social or communicative problems himself; meaningful social contacts are limited, even with family members and colleagues. He had a normal development. His medical history includes an inguinal hernia and hydrocele in childhood, both of which were surgically corrected. The family history does not mention any medical or developmental problems. At physical examination, no typical facial characteristics were observed.

Subject 2 is a 43-year-old female, mother of two children, and her youngest daughter was diagnosed with KS. The somatic characteristics of subject 2 and her youngest daughter have been previously described, represented as family 2 [3]. At the age of 13 years, the subject emigrated from Morocco to the Netherlands. She experienced learning difficulties and did not finish high school. Besides running the household, she started working as a care assistant for elderly people. She was not able to take care of her own financial administration, because of learning difficulties. Her psychiatric history reported a MDD during pregnancy for which fluoxetin was prescribed. Further medical history mentioned three spontaneous abortions, psoriasis, and nephritis. Additionally, she experiences sleep disturbances comprising apneas and frequent awakenings during the night.

Her social network was limited to her family. She had no friends and experienced problems in initiating and maintaining contacts and conversations. Small talk was difficult for her. At the time of examination, she was divorced and not able to work due to mental health problems, consisting of problems in social reciprocity, reactive depressive complaints, and overstimulation and burnout.

At the time of our investigations, she experienced a second major depressive episode with psychotic features (imperative hallucinations and delusions). The imperative hallucinations had led to a suicide attempt. Her general practitioner was informed after our examination, and intensive outreaching care was suggested based on our observation. Physical examination of this subject at the age of 43 years showed obesity and minor dysmorphic features as previously described, including a hypoplastic midface, small upslanting palpebral fissures, depressed nasal root, and anteverted nares.

Subject 3 is a 39-year-old male with three children: the second child was diagnosed with KS. There is no further family history of somatic or neuropsychiatric disorders. After finishing primary school, he struggled to finish high school (practical education level) because of learning disabilities. He went through different jobs, but at the time of the current evaluation, he was not able to work due to his impaired somatic and mental health. He lives an isolated life, together with his wife and children. Despite his social isolation, the patient did not experience any problems in social behavior.

His birth was complicated by a forceps delivery. The remaining medical history revealed atrial fibrillation, knee surgery, tonsillectomy, tympanostomy, and mild auditory and visual impairment. Pharmacotherapy started after the atrial fibrillation was diagnosed and comprised the following: metoprolol, acenocoumarol, simvastatin, and perindopril. Physical examination at the age of 39 years showed obesity, an occipito-frontal circumference of 59 cm (nearly + 1 SD), and minor dysmorphic features including a hypoplastic midface and synophrys.

### Genetic analysis

Subject 1: After the detection of an intragenic *EHMT1* deletion in the son by routine array analysis, we performed an MLPA (kit P340-A1, MRC-Holland, Amsterdam, The Netherlands) analysis to confirm the deletion in subject 1. We detected an intragenic deletion of exons 5–17 of *EHMT1* in DNA from both blood lymphocytes and a buccal swab sample, with a mosaicism of 40%. In cultured fibroblasts, the level of mosaicism was 15–20% of cells carrying the deletion.

Subject 2: Results were presented in more detail in a previous study [3]. In summary, genome-wide array analysis on DNA from the daughter revealed an interstitial deletion of ~ 200 kb in 9q34.3 encompassing at least the first five exons of the *EHMT1* gene. Carrier testing by array in both parents showed that this deletion was inherited from subject 2 (the mother), but her array results showed a less pronounced loss, suggestive of mosaicism.

Interphase FISH analysis with two different FISH probes on a buccal swab sample from subject 2 showed the deletion in 80% of her cells (50–55 cells analyzed), indicating that she is a mosaic carrier of the 9q34.3 deletion: .nuc ish del(9)(q34.3q34.3)(RP11_417A4-)[37/55],(RP13_467E5-)[45/50].

Subject 3: After the detection of a 9q34.3 deletion in the son (arr [hg 19] 9q34.3(140350924 140551108)× 1), the array analysis on DNA from subject 3 suggested the presence of a mosaic deletion in 9q34.3. Subsequent FISH analyses on blood lymphocytes showed an abnormal signal pattern in 29 of 50 cells analyzed, suggesting 60% mosaicism: ish del(9)(q34.3q34.3)(RP11-48C7-)[29/50].

## Results

The genetic, psychopathological, and neurocognitive characteristics are presented in Table 1.

### Psychopathological and neurocognitive studies

The developmental age of all three mosaic carriers was 12 years or older. This maximum score on the clinical interview VABS is in line with the developmental age of the general population. Additionally, all subjects fulfilled the diagnostic criteria for ASD and MDD either currently and/or in the past. In contrast to the ADOS, the mini PAS-ADD did not formally determine ASD as the total subscale scores were just below the cutoff score. Notwithstanding, ASD items scored in the mini PAS-ADD were similar to the clinical observations of the ADOS. During the clinical observation, all participants scored on limited eye contact and minimum use of emotional gestures, when testing was focused on the communication. For social interaction, all three showed impaired social reciprocity, a limited range of facial expressed emotions, and an inadequate description of their role in social relations.

In addition to estimating the degree of psychopathology, we used the CANTAB to measure several cognitive functions of our subjects. The percentages represent the subjects’ performance compared to the normative group with the same sex and age. Subject 1 had low scores on the PRM and IED. Subject 2 had overall low scores on the tests. Subject 3 experienced problems with the IED. Detailed results are presented in Table 1; all IED scores were lower than 35% of the peer group, indicating an impaired mental flexibility in all three subjects.

## Discussion

In this report, we describe the consequences of *EHMT1* mosaicism on psychopathology in three adult subjects. The three seemingly unaffected parents included in this study had been referred to the clinical genetics department only because of the diagnostic trajectory of each parent’s respective child. The discovery of *EHMT1* mosaicism in these subjects was unexpected [3–5]. The degree of mosaicism that was measured in different tissues varied from 15 to 40% (subject 1) to 80% (subject 2). It is difficult to predict the clinical consequences of mosaicism, as the patterns and distribution of abnormal cells can vary widely between different tissues, depending on the timing of the mutation events [20]. Despite this predicted variation, all three subjects had an ASD, a (prior) MDD, and other associated neurocognitive dysfunctions. Some features that can be seen in patients with a germline *EHMT1* mutation had a variable occurrence in the mosaic individuals. The results are indicative of a vulnerability to develop severe psychiatric disorders that carriers of an *EHMT1* mosaic mutation possess. This correlates with the high prevalence of psychopathology, which has been reported for individuals with the full KS phenotype due to a germline *EHMT1* mutation [6, 8, 21]. Based on the ADOS test, all mosaic subjects met the criteria for ASD and all had a (prior) MDD. This is further corroborated by our findings on the CANTAB. All subjects showed weaknesses in performance on the IED, which is suggestive of cognitive inflexibility. This might be related to the set-shifting problems often seen in subjects with ASD, although findings on executive function deficits in adults with ASD were inconsistent [22]. These deficiencies on the IED are on the continuum compared with a significant high dropout on the IED in our previous research on subjects with the full KS phenotype (unpublished data). Subject 3 had no other deviating scores compared to his norm group. Subject 1 showed performance problems on the PRM, but we could not clearly link this to his clinical profile. Subject 2 had low scores on all tasks, most likely due to (a combination of) her learning and mood problems.

Psychopathology has a direct impact on adaptive functioning. The adaptive behavior measured with the VABS implies a developmental age above 12 years, consistent with adult functioning, for all of our subjects. Nevertheless, all three of them struggle to fulfill their role in society, most likely as a direct consequence of their neurodevelopmental problems. They became socially isolated and experienced difficulties in finding the right type of education and employment that would suit their strengths and weaknesses. Therefore, the clinical relevance for diagnosing these subjects with *EHMT1* mosaicism and consequent neuropsychiatric assessment is significant. Optimal treatment of the psychopathology may improve daily functioning and positively influence the well-being of the individuals treated and their families, including the child with KS, as well as their participation in society. Based on our findings, personalized psychiatric evaluation and treatment should be considered for subjects diagnosed with *EHMT1* mosaicism. The diagnostic procedure should include a special attention to ASD and mood disorders. Furthermore, these cases illustrate that presence of mosaic deletions could potentially be missed, since the genetic defects were only discovered during the diagnostic phase of the children with the full KS phenotype. None of the subjects had been referred to a medical specialist for their own psychiatric problems prior to our studies. These subjects tend to have a more subtle presentation, but this can nevertheless cause impaired (social) functioning. Therefore, our study also demonstrates that genetic analysis should be considered in patients with a psychiatric disorder and other characteristics of KS without the classic appearance.

Despite the similarities in psychopathology between our subjects, the consequences of mosaicism in general are widespread and unique for each individual [23]. The phenotypic expression of somatic mosaicism has been investigated and reported in several studies including mosaic trisomy 21 (Down syndrome) and mosaic fragile X syndrome [24, 25]. These studies show severe phenotypic expression, as well as somatic mosaicism without clinical consequences. The expression of the behavioral phenotype is also variable. In mosaic trisomy 21, for example, the social adaptive skills were significantly better in the subjects with a mosaicism compared to non-mosaic subjects with Down syndrome. However, the intellectual performance did not relate to the level of mosaicism [24]. This necessitates interpretative caution, especially since we assessed the psychopathology of only three subjects with an *EHMT1* mosaicism and each had different levels of mosaic expression. Interestingly, in recent studies, it was shown that rare postzygotic mutations in important developmental genes contribute significantly to ASD [12, 13]. Our present results obtained for three ASD subjects with such a rare mosaic defect in *EHMT1* are in line with these reports. *EHMT1* is an important developmental gene that is extremely intolerant to loss-of-function mutations (http://exac.broadinstitute.org) [26]. Further clinical studies are warranted to provide more insight into the wide spectrum of the *EHMT1* mosaicism, although the extremely low prevalence of such mosaic defects hampers the careful collection of symptoms in larger cohorts. For future research, we recommend to include more specific measures on IQ, ASD, and mood disorders in subjects with an *EHMT1* mosaicism. These measures should be in addition to a (semi)structured clinical observation as people with neurodevelopmental disorders, like ASD, are more likely to have deviating scores on questionnaires [27, 28]. Thus, clinical interviews and observations should always be performed to gain a more comprehensive and objective insight into the psychopathology.

## Conclusion

Subjects with *EHMT1* mosaicism seem vulnerable to developing psychiatric disorders, especially ASD and mood disorders. However, since the behavioral outcome of mosaicism is unpredictable, these results should be interpreted with caution. Based on our findings in this small but unique cohort, we recommend screening subjects with an *EHMT1* mosaicism for psychopathology. In our experience, multidisciplinary care, combining expertise in psychiatry, clinical genetics, and clinical neuropsychology, is of additional value in these complex cases where a broad perspective is required. Ideally, a careful diagnostic assessment of psychopathology, cognition, and physical health should be performed to provide an integrative diagnosis. Deficits in daily functioning and behavioral problems may well be a result of an underlying genetic or otherwise somatic cause, which is often not clearly present. Clinical considerations should therefore include the possibility of a genetic mosaicism in parents who have one or more children with a neurodevelopmental disorder of genetic origin and a typical combination of complaints, as illustrated in this study.

## Abbreviations

ADOS: Autism Diagnostic Observation Schedule
ASD: Autism spectrum disorder
CANTAB: Cambridge Neuropsychological Test Automated Battery
EHMT1: Euchromatin histone methyltransferase 1
ICD-10: International Classification of Diseases-10
ID: Intellectual disability
IED: Intra-Extra Dimensional Test Shift
KS: Kleefstra syndrome
MDD: Major depressive disorder
MOT: Motor Screening Test
PAL: Paired Associate Learning
PAS-ADD: Psychiatric Assessment Schedules for Adults with Developmental Disabilities
PRM: Pattern Recognition Memory
PZM: Postzygotic mutations
VABS: Vineland Adaptive Behavior Scales
